# Impact of the COVID-19 Pandemic on the Severity of Diabetic Ketoacidosis Presentations in a Tertiary Pediatric Emergency Department

**DOI:** 10.1097/pq9.0000000000000502

**Published:** 2022-03-30

**Authors:** Kaileen Jafari, Ildiko Koves, Lori Rutman, Julie C. Brown

**Affiliations:** From the Seattle Children’s Hospital, University of Washington.

## Abstract

More severe presentations of diabetic ketoacidosis (DKA) have been reported during the coronavirus disease 2019 (COVID-19) pandemic, possibly due to avoidance of healthcare settings or reduced access to care. To date, no studies have utilized statistical process control to relate temporal COVID-19 events with DKA severity. Our objectives were (1) to determine whether the severity of pediatric DKA presentations changed during COVID-19 and (2) to temporally relate changes in severity with regional pandemic events.

**Methods:**

This study was a retrospective chart review of 175 patients younger than 18 years with DKA presenting to a pediatric emergency department in the United States between 5/1/2019 and 8/15/2020. As part of our ongoing clinical standard work in ED management of DKA, DKA severity measures, including presenting pH, the proportion of PICU admissions, and admission length of stay, were analyzed using statistical process control.

**Results:**

During COVID-19, we found special cause variation with a downward shift in the mean pH on DKA presentation from 7.2 to 7.1 for all patients. The proportion of DKA patients requiring PICU admission increased from 34.2% to 54.6%. Changes temporally corresponded to the statewide bans on large events (3/11/2020), school closures (3/13/2020), and a reduction in our institution’s emergency department volumes. Admission length of stay was unchanged.

**Conclusions:**

Pediatric DKA presentations were more severe from March to June 2020, correlating with regional COVID-19 events. Future quality improvement interventions to reduce delayed presentations during COVID-19 surges or other natural disasters should target accessibility of care and public education regarding the importance of timely care for symptoms.

## INTRODUCTION

The coronavirus disease 2019 (COVID-19) pandemic resulted in significant reductions in emergency department (ED) utilization both within the United States and abroad.^1–6^ Estimates from the Centers for Disease Control indicate that during April 2020, national ED utilization decreased by 42% compared with in prior years, with the most significant reductions in utilization seen in patients younger than 14 years of age.^1^ A recent report from our institution’s pediatric ED noted reducing ED volumes of up to 72% during March–April 2020 compared with similar months in prior years.^4^ A growing body of evidence suggests reduced utilization stems from public fears of contracting COVID-19 in healthcare settings.^2–6^ Delays in timely care for emergent conditions have led to increased illness presentation severity and increased disease complications.^5–8^

Diabetic ketoacidosis (DKA) is a condition sensitive to timely diagnosis, with increased severity noted in those with new-onset diabetes, initial misdiagnosis, and limited access to care.^9–13^ Multiple retrospective studies from Europe and Australia reported pediatric DKA presentations to be more severe during the initial months of the COVID-19 pandemic than in similar months during prior years.^14–18^ This American study is the first to relate temporal regional events and public health policies of the COVID-19 pandemic with the severity of DKA presentations in children, using statistical process control to evaluate and assess system changes over time. This study aimed to explore the association between regional events, public health policies, and associated changes in public attitudes during the COVID-19 pandemic with the severity of DKA presentations in pediatric patients, with the ultimate goal of identifying quality improvement interventions to reduce DKA severity during future COVID-19 surges.

## METHODS

### Context

The setting is a free-standing tertiary pediatric referral center located in Seattle, Washington. The hospital ED has 50,000 ED visits annually, with 20% of encounters resulting in admission. This pediatric hospital is the only regional facility providing inpatient pediatric endocrinology services and cares for most pediatric DKA patients in Seattle and the surrounding region. As part of the institution’s model of continuous process improvement, all patients with DKA in the ED are managed on the DKA clinical standard work (CSW) pathway.^19^ The DKA CSW team monitors numerous DKA ED care metrics, allowing for frequent assessments of the quality of ED DKA care and implementing targeted interventions to improve care. This study was conducted as a sub-analysis of the DKA CSW pathway as part of ongoing ED quality and safety efforts.

### COVID-19 in the Seattle Region

**Fig. 1.**
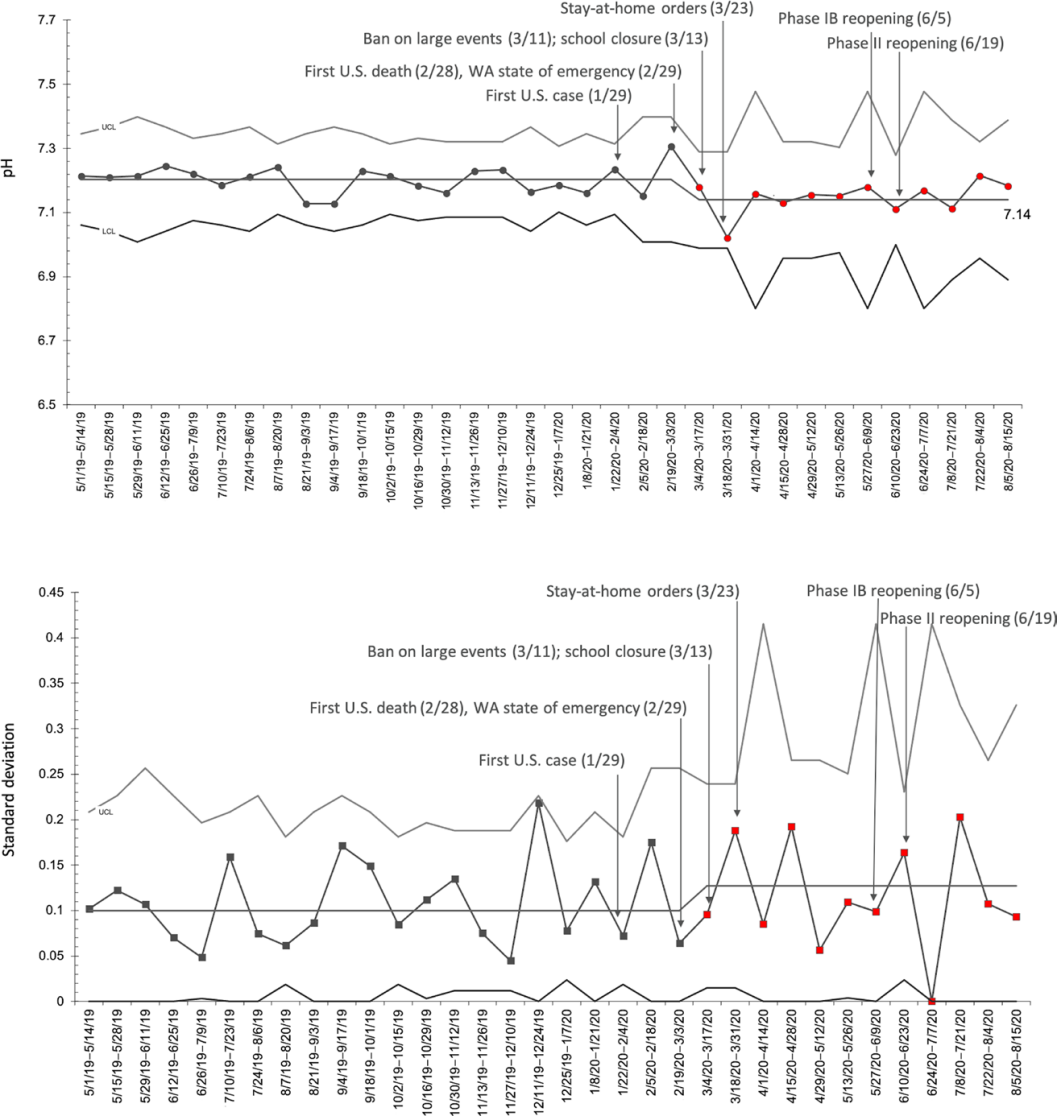
X-bar-S Chart: mean and SD of presenting pH for DKA patients. A plot of the average biweekly presenting pH of DKA patients from May 1, 2019 to August 15, 2020. Annotations indicate the key regional events during the COVID-19 pandemic. The period of 3/4/2020 through 8/15/2020 meets the criteria for special cause variation.

The Seattle region was among the first locations in the United States impacted by COVID-19. A detailed timeline of events can be seen in Supplemental Digital Content 1. (**See figure 1, Supplemental Digital Content 1,** which shows the Timeline of COVID-19 events in the Seattle region. A timeline of key events, including the first cases of COVID-19 and pandemic-related public health policies, is noted for the Seattle region. http://links.lww.com/PQ9/A334) The first U.S. case of COVID-19 was confirmed on January 2, 2020, near Seattle, Washington. The Seattle region reported the first U.S. death from COVID-19 on February 29, 2020, at a long-term care facility, which is subsequently associated with an outbreak resulting in 23 deaths.^20^ Initial social distancing policies were implemented starting 3/11/2020, followed by a stay-at-home policy on 3/23/2020.^21^ Our institution transitioned to primarily tele-visits for ambulatory subspecialty appointments during the first 2 weeks of March 2020. Many outpatient pediatric practices also transitioned to telemedicine, and reduced in-person well-child visits in late March. Phased community re-opening in the Seattle region started on 6/5/2020.^21^

### Study Design

This report is of a retrospective chart review of pediatric patients presenting with DKA to the ED between May 1, 2019, and August 15, 2020. The timeframe included 10 months before and 5.5 months after the initial COVID-19 events in our region. Patients younger than 18 years of age with an ICD10 discharge diagnosis of DKA (ICD10 codes E10.1, E08.1 E09.1, E11.1) were included. We included all patients treated for DKA in our ED, including those who presented initially to an outside facility and then transferred to our ED for further care (ED transfers). We planned to exclude patients for whom initial pH at their first hospital presentation was not available; however, no patients met this exclusion criterion.

### Measures

This study’s primary objective was to assess the impact of COVID-19 on the severity of pediatric DKA presentations in the ED. The primary outcome measure was pH at the time of presentation to the ED (presenting pH). We used presenting pH at the outside facility before treatment for ED transfers. We selected presenting pH as the primary outcome measure based upon the International Society for Pediatric Diabetes DKA guidelines, which characterize DKA severity based upon presenting pH.^22^ Secondary outcomes included other measures of DKA severity, including pediatric intensive care unit (PICU) admission, glucose on presentation (presenting glucose), administration of osmotic fluids (hypertonic saline or mannitol) as a proxy for cerebral edema, mechanical ventilation, and inpatient length of stay.

### Analysis

We used descriptive statistics to compare the demographics and clinical characteristics of the pre-COVID-19 (May 1, 2019–February 29, 2020) and COVID-19 (March 1, 2020–August 15, 2020) groups. We used Mann-Whitney to compare continuous variables and Chi-Square/ANOVA to compare categorical variables. In addition, we used statistical process control (SPC) to analyze the following measures: average presenting pH, average presenting blood glucose level, hospital LOS (days), and PICU admission percentage. SPC charts are useful for monitoring system-level data over time and permit differentiation of normal variation inherent to a process, known as common cause variation, from variation due to an assignable cause, known as special-cause variation.^23^ We chose to utilize SPC as a tool to explore the temporal relationship between specific pandemic events and the severity of DKA presentations. Standard SPC rules were used to define special cause variation.^23^ SPC charts were annotated with the key events of the COVID-19 pandemic in the Seattle region to correlate them with changes in DKA severity. We used QI Charts 2.0 add-in for Microsoft Excel (Process Improvement Products, Austin, TX) to create all SPC charts.

Additional secondary outcomes that were not amenable to meaningful SPC analyses were analyzed using pre- and post-analysis. These included osmotic fluid administration and mechanical ventilation compared between pre-COVID-19 and COVID-19 groups using Fischer’s exact test. Additionally, we compared the mean monthly number of DKA presentations for patients with established and new-onset diabetes using a *t* test and attribute charts (c-charts). Small sample sizes in the subset analysis for patients with new-onset diabetes prevented meaningful SPC analysis for outcomes other than presenting pH. We compared these secondary outcomes, including proportion PICU admission using chi-square test and osmotic fluid administration using Fischer’s exact test. STATA (StataCorp, LLC, College Station, Tex.) was used for these analyses.

### Ethical Considerations

This study was reviewed and approved by the institution’s Institutional Review Board, which approved waiver of informed consent.

## RESULTS

Between May 2019 and August 2020, 175 patients were treated in the ED for DKA: 119 pre-COVID-19 and 56 after the onset of COVID-19. All 175 patients met the inclusion criteria. There was an average of 11.9 DKA presentations per month pre-COVID-19 and 10.2 DKA presentations per month in the COVID-19 group. The pre-COVID-19 and COVID-19 groups were similar in age, sex, initial presenting ED location, and preferred language of care (Table 1). There were fewer non-White patients in the COVID-19 group compared with the pre-COVID-19 group (*P* value = 0.02). Patients with new-onset diabetes represented significantly more of the COVID-19 group compared with pre-COVID-19 (60.7% versus 40.5%, *P* value = 0.046).

**Table 1.** Demographic and Visit Characteristics of all Pediatric DKA Patients

	Pre-COVID-19 (n = 119)	COVID-19 (n = 56)	All Patients (n = 175)	*P*
Age, Median (IQR)	12 (8–15)	12 (8.5–13.5)	12 (8–15)	0.410
Women, n (%)	63 (52.9%)	27 (48.2%)	90 (51.4%)	0.341
Type 1 DM, n (%)	119 (100%)	56 (100%)	175 (100%)	1.0
New onset diabetes, n (%)	53 (44.5%)	34 (60.7%)	87 (59.7%)	0.046*
Initial presentation at our ED, n (%)	91 (76.5%)	42 (75.0%)	133 (76.0%)	0.832
Language, n (%)				
English	103 (86.6%)	54 (96.4%)	57 (89.7%)	0.133
Spanish	7 (5.9%)	1 (1.8%)	8 (4.6%)	
Other	9 (7.6%)	1 (1.8%)	10 (5.7%)	
Race/ethnicity, n (%)				
White	71 (59.7%)	43 (76.8%)	114 (65.1%)	0.021*
Black	11 (9.2%)	0 (0%)	11 (6.3%)	
Other	37 (31.1%)	13(23.2%)	50 (28.6%)	
COVID-19 testing, n (%)				
Performed	0 (0%)	51 (91.1%)	51 (29.1%)	
Positive	N/A	0 (0%)	0 (0%)	

*P < 0.05.

DM: Diabetes Mellitus; IQR: Interquartile range.

Results of COVID-19 Polymerase Chain reaction (PCR) testing (Center for Disease Control approved SARS CoV-2 qPCR assay (Integrated DNA technologies, Iowa, USA)) were available for 51/56 patients in the COVID-19 group (91.1%, see Table 1). All results of COVID-19 PCR testing were negative. The monthly number of new-onset diabetes DKA presentations remained stable throughout the study period. (**See figure 3, Supplemental Digital Content 4,** which shows the C-chart: monthly counts of DKA presentations among patients with new-onset and established diabetes. We plotted the count of DKA presentations for those with established and new-onset diabetes from May 1, 2019 to August 15, 2020. There were fewer established DKA presentations during the months of February to August 2020 (circled in red); however, there were insufficient timepoints to meet special cause variation. http://links.lww.com/PQ9/A337.) In contrast, the pattern in the established diabetes c-chart suggests an overall decrease in DKA presentations during the pandemic period. However, there were insufficient timepoints to meet special cause variation criteria.

### Presenting pH of DKA Patients

The presenting pH of DKA patients and its temporal relationship to various local events in the COVID-19 pandemic is shown on the annotated SPC chart (Fig. 1). Special cause variation occurred starting from 3/4/2020 to 3/17/2020, with a shift of less than 8 consecutive points below the centerline. This special cause variation correlates temporally with the statewide bans on large events (3/11/2020) and the closure of schools (3/13/2020). This biweekly period also corresponds to overall reductions in ED utilization within our institution, as published previously.^4^ Lower presenting pH persisted through the end of June 2020, after which data trended back toward baseline levels. On the corresponding S-chart, special cause variation was also noted during the weeks of 3/4/2020–3/17/2020, with an upward shift suggesting that there was a greater variability in presenting pH during this time compared with the baseline.

**Fig. 2.**
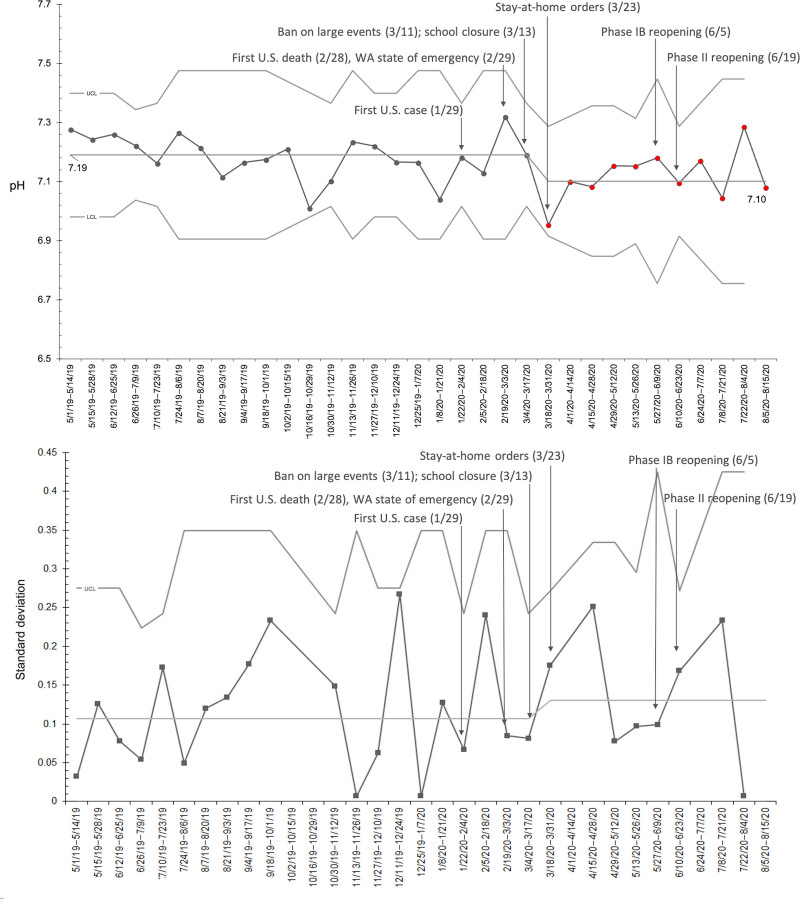
X-bar/sec-Chart: mean and SD of presenting pH for DKA patients with new-onset diabetes. We plotted the average biweekly presenting pH of DKA patients with new-onset diabetes from May 1, 2019 to August 15, 2020. Annotations indicate the key regional events during the COVID-19 pandemic. The period 3/18/2020–8/15/2020 meets the criteria for special cause variation.

A sub-analysis of patients with new-onset diabetes yielded similar results (Fig. 2). We treated a total of 87 patients with new-onset diabetes for DKA during the study period, 53 in the baseline period, and 34 patients during the COVID-19 period. A decrease in presenting pH meeting criteria for special cause variation occurred between 3/18/2020 and 3/31/2020 (Fig. 2). Data from April 2020 through the end of July 2020 indicate that these effects were persistent, with a subsequent trend toward baseline. A sub-analysis of patients with established diabetes demonstrated a trend of lower pH during the pandemic period; however, it did not meet the criteria for special cause variation.

### PICU Admissions for DKA Patients

**Fig. 3.**
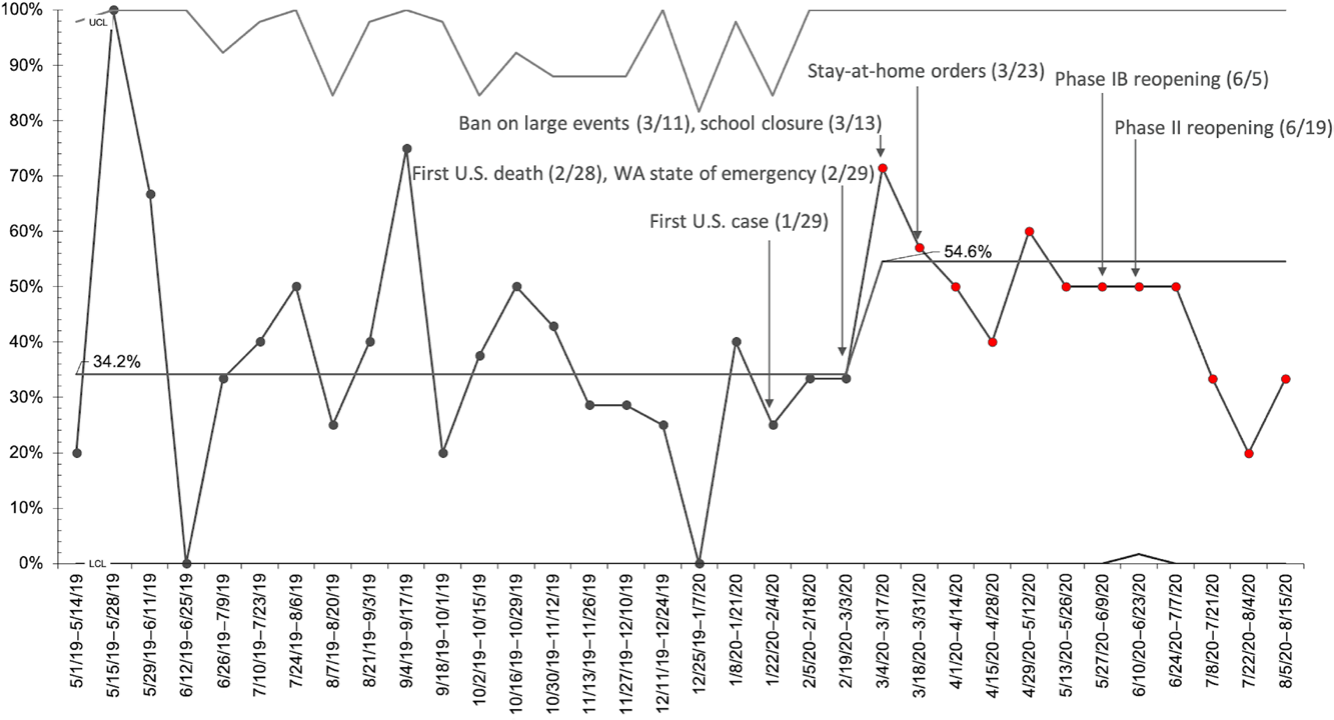
*P*-chart: proportion of DKA admissions requiring PICU care. We plotted the biweekly proportion of DKA admissions requiring PICU care from May 1, 2019–August 15, 2020. Annotations indicate key regional events in the COVID-19 pandemic. The period of 3/4/2020–3/17/2020 through 8/15/2020 meets the criteria for special cause variation.

The annotated SPC chart in Figure 3 shows the proportion of DKA patients admitted to the PICU and the temporal relationship of PICU admission to various local events in the COVID-19 pandemic. Our institution utilizes a standardized set of criteria as part of our CSW pathway to determine intensive care needs for DKA patients. These criteria include initial pH (<7.15), initial HCO_3_ *<*5 mEq/L, and age younger than 24 months, among others (https://www.seattlechildrens.org/pdf/DKA-pathway.pdf). No changes to the admission criteria were made during the study period. During the baseline period of May 2019 to February 2020, a total of 40 patients with DKA required PICU admission, representing 33.6% of total admissions (Fig. 3). In total, 23 of these patients had new-onset diabetes (57.5%), and 17 had established diabetes (42.5%). An increased proportion of PICU admissions (54.6%) was noted during the weeks of 3/4/2020–3/17/2020, meeting criteria for special cause variation and coinciding directly with Washington state’s ban on large events (3/11/2020) and school closures (3/13/2020). These effects persisted through June 2020, after which we observed a trend toward the pre-COVID baseline proportion of PICU admissions.

### Secondary Outcomes

The annotated SPC chart in Supplemental Digital Content 3 shows inpatient length of stay. (**See figure 2, Supplemental Digital Content 3,** which shows the X-bar/sec-chart: Mean and SD of length of stay for DKA patients. We plotted the mean biweekly length of stay of DKA patients from May 1, 2019 to August 15, 2020. Key regional events in the COVID-19 pandemic are annotated. Criteria for special cause variation are met starting 3/18/2020–8/15/2020. http://links.lww.com/PQ9/A336.) Throughout the study period, the average length of stay was 2.0 days. Therefore, the X-bar chart did not meet the criteria for special cause variation. However, in the corresponding S-chart, a decrease in the SD during the weeks of 3/4/2020–3/17/2020 did meet special cause variation.

The mean number of monthly DKA presentations for new-onset patients and established patients was not significantly different between pre-COVID-19 and COVID-19 groups (Table 2). Although some measures of severe DKA, including osmotic fluid administration and mechanical ventilation, occurred in higher frequency in the COVID-19 group, these were very infrequent events, and differences were not statistically significant. In the COVID-19 group, COVID-19 PCR was performed in 51/56 patients; all patients were negative (see Table 1). For the subset analysis of patients with new-onset diabetes only, there was a trend of increased PICU admissions, osmotic fluid (mannitol or hypertonic saline) administration, and mechanical ventilation in the COVID-19 group; however, these differences were not statistically significant. (**See table, Supplemental Digital Content 2,** which shows the secondary outcomes for pediatric patients with new-onset diabetes presenting with DKA. http://links.lww.com/PQ9/A335).

**Table 2.** Secondary Outcomes for all Pediatric Patients with DKA during Pre-COVID-19 and COVID-19 Time Periods

	Pre-COVID-19 (n = 119)	COVID-19 (n = 56)	All Patients (n = 175)	*P*
New-onset DKA presentations/mo, mean (SD)	5.3 (3.82–6.78)	5.7 (2.37–8.96)	5.44 (4.15–6.71)	0.781
Established DKA presentation/mo, mean (SD)	6.6 (4.23–8.97)	3.67 (2.58–4.75)	5.5 (3.9–7.1)	0.056
Osmotic fluid administration,^*^ n (%)	3 (2.5%)	3 (5.5%)	6 (3.4%)	0.386
Mechanical ventilation, n (%)	0 (0%)	2 (3.6%)	2 (1.1%)	0.101

*Osmotic fluid administration: Mannitol or hypertonic saline.

## DISCUSSION

This study is the first to confirm increased DKA severity in pediatric patients in the United States during COVID-19 and to utilize SPC analysis to determine the temporal relationship between regional COVID-19 events and the severity of DKA presentations. Our findings are congruent with reports from Europe and Australia, which identified increased frequency and severity of pediatric DKA presentations during the COVID-19 pandemic.^13–18^ A survey of pediatric consultants from the United Kingdom reported DKA as the most frequent condition subject to delayed diagnosis in children in the ED/urgent care setting during the COVID-19 pandemic.^25^ Similarly, in multiple retrospective analyses from Europe and Australia, the COVID-19 pandemic was associated with significant increases in the frequency and severity of DKA presentations in pediatric patients compared with prior years.^13–18^

The timing of special cause variation occurred several weeks following an extensive community outbreak of COVID-19 and during statewide bans on large gatherings (3/11/2020) and school closures (3/13/2020). This period also temporally corresponded to a 72% reduction in our institution’s ED volumes.^4^ We hypothesize delayed presentation to care as the primary driver of the increased severity in DKA, consistent with prior studies demonstrating increased DKA severity in those with initially missed diagnosis and limited access to care.^12^ We theorize public fears of COVID-19 combined with COVID-19-related reductions in access to care were likely responsible for these delays in presentation, ultimately resulting in increased severity of DKA presentations. This possibility may be particularly relevant for those with new-onset diabetes, as there appeared to be a trend of fewer DKA presentations among established diabetics during the pandemic period. Special cause variation in DKA severity persisted through at least early July 2020, followed by a subsequent trend toward the baseline.

**Fig. 4.**
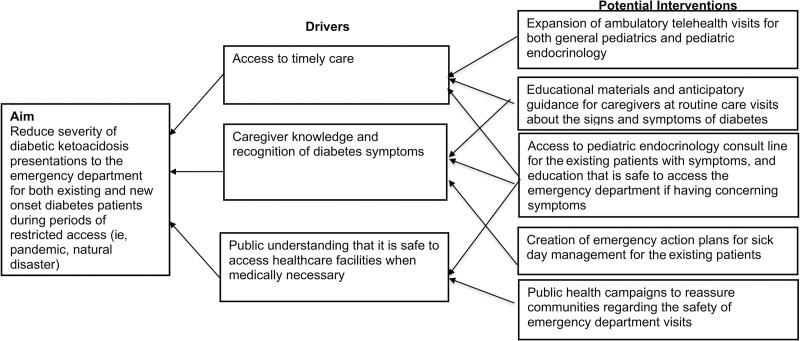
Key driver diagram. Proposed key driver diagram to reduce delayed diabetes presentations during future pandemics or natural disasters with restricted health care access.

This study was performed as a sub-analysis of ongoing efforts to monitor and iteratively improve our DKA pathway. Our findings emphasize the potential role of quality improvement (QI) interventions beyond routine care standardization to reduce delayed presentations during future pandemic surges or other natural disasters that limit access to care. Accordingly, we created a key driver diagram to outline potential QI interventions to reduce delayed presentations (Fig. 4).

We identified three primary drivers of delayed presentations: (1) access to timely care, (2) caregiver knowledge and recognition of diabetes symptoms, and (3) public understanding that it is safe to access healthcare facilities when medically necessary. Access to timely care could be addressed through expansion and improvements in existing telehealth from both primary care and pediatric endocrinology services. To address the key driver of caregiver knowledge, QI teams should outreach to primary care providers to (1) increase awareness about delayed presentations in children; (2) encourage screening for symptoms of diabetes at every visit and; (3) provide education to families about concerning signs and symptoms of diabetes is needed. For existing patients with diabetes, recent COVID-19 International Society for Pediatric Diabetes guidelines recommend enhanced education of families around sick day management. Specific interventions include (1) review of sick day management with caregiver/patient teach-back at every pediatric endocrinology visit and (2) creating emergency action plans tailored to COVID-19 conditions, including when and where to access emergency care.^27^ Our final key driver, public understanding that it is safe to access healthcare facilities when medically necessary, could be addressed through public health campaigns to bring awareness to the issue of delayed presentation and to emphasize the importance of seeking care when medically necessary.

The differences noted in racial distribution between the pre-COVID19 and COVID-19 groups may suggest that certain vulnerable populations may be at a higher risk of delayed presentations with DKA. A recent publication from the Centers for Disease Control reported that 4 in 10 adults delayed care due to COVID-19 fears, with increased rates of delayed care in patients with disabilities, chronic illness, and Black and Hispanic adults.^28^ Additionally, prior research has identified racial minorities and those with low socioeconomic status to be at a higher risk of more severe DKA.^11,28^ Families with limited computer access and lower health literacy may find access to telemedicine challenging, further reducing disparities in care. Ongoing research to identify potential disparities in COVID-19 associated delayed presentations and access to care for pediatric patients during pandemic surges is needed.

Our study has several limitations. First, this was a single-center study in a region of the United States which had the earliest cases of COVID-19. Thus, our findings may not apply to other regions or different periods during the COVID-19 pandemic. Furthermore, unmeasured factors may have contributed to changes in DKA severity. For example, we noted a trend of fewer established diabetes DKA presentations during the pandemic period. This observation could indicate that some patients with established diabetes and mild DKA may have successfully treated their DKA at home and avoided the ED, resulting in a relative skewing of our DKA severity measures.^29^ Our primary outcome, pH on presentation, is also a limitation because this measure could vary depending on the laboratory and method used to calculate pH.

Additionally, increased PICU bed availability during the pandemic may have resulted in more DKA admissions to the PICU, thus increasing the PICU admission rates. Because this was a retrospective chart review, we could not determine if families intentionally delayed care due to fear of healthcare settings or limited access to care resulted in delayed diagnosis. Access to care limitations, including transitions to telemedicine for primary care and endocrinology, and reduced well-child care in the outpatient setting may have further contributed to delayed diagnoses. Finally, although we saw a trend toward baseline in presenting pH and PICU admissions in early July, corresponding to initial re-opening phases in the region, there were insufficient timepoints to meet special cause variation criteria.

## CONCLUDING SUMMARY

DKA severity, as assessed by presenting pH, worsened after the start of the COVID-19 pandemic. Similarly, intensive care admission, an indirect proxy for DKA severity, also worsened during the COVID-19 pandemic. There was a temporal association between regional events related to the pandemic and measures of DKA severity. The effects met special cause 2 months after the pandemic’s start as regional social distancing policies came into effect, and they were persistent for at least 3 months. This work emphasizes the need to develop strategies for managing chronic health conditions in pediatric patients during unexpected interruptions in normal care, such as those imposed by the COVID-19 pandemic and other natural disasters. Implementation of QI interventions targeting key drivers of delayed access to care may be a useful first step in this process. Further research is needed to identify particularly vulnerable patients for delayed diagnosis, strategies to prevent delays in care, and the duration of the effects of delayed presentations.

## DISCLOSURE

The authors have no financial interest in relation to the content of this article.

## ACKNOWLEDGMENTS

We thank the following team members for their assistance with the study: Anne C. Slater, MD, Assistant Clinical Professor, Seattle Children’s Hospital; James B. Johnson, Clinical Effectiveness Analyst, Seattle Children’s Hospital; and Nathan Deam, Clinical Analytics Manager, Seattle Children’s Hospital.

## Supplementary Material


