# Supplementary figures and images for: Impact of the COVID-19 Pandemic on the Severity of Diabetic Ketoacidosis Presentations in a Tertiary Pediatric Emergency Department
Source: Pediatr Qual Saf. 2022 Mar 30;7(2):e502. doi: 10.1097/pq9.0000000000000502 (PMC8970094; doi:10.1097/pq9.0000000000000502)

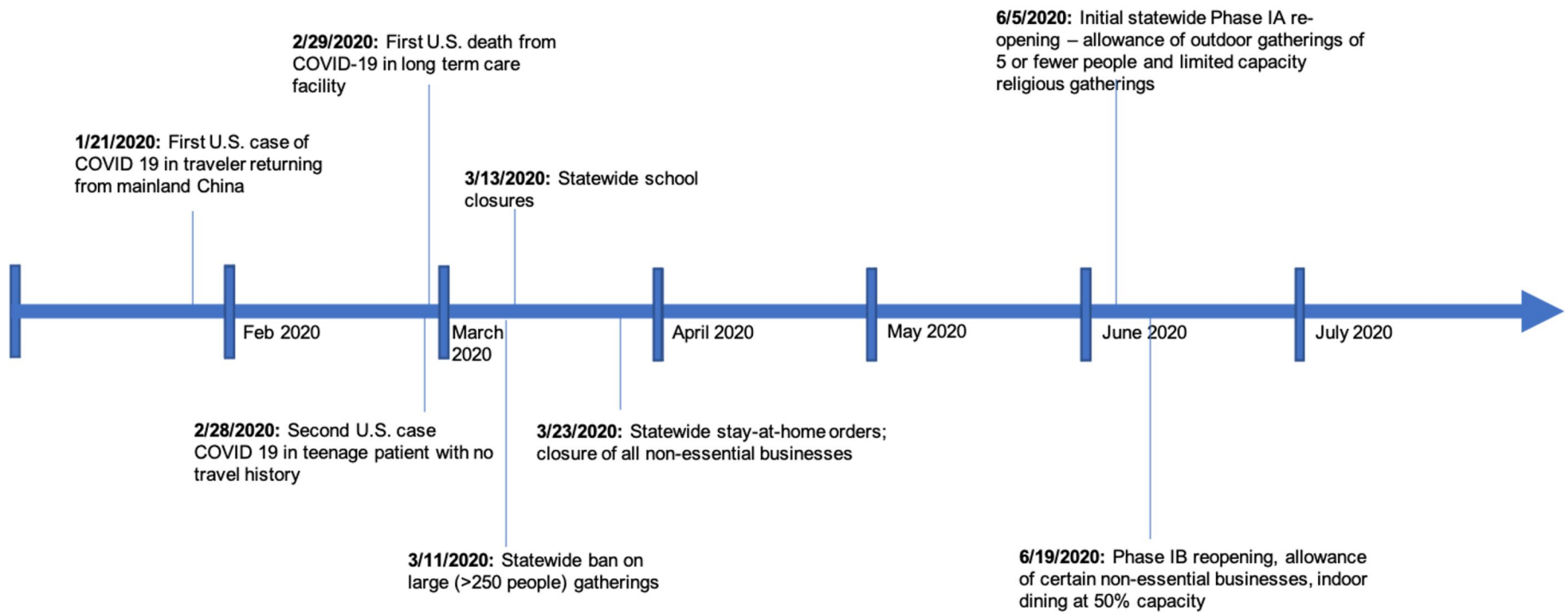

Supplement: Supplementary file 1 [file pqs-7-e502-s001.pdf]

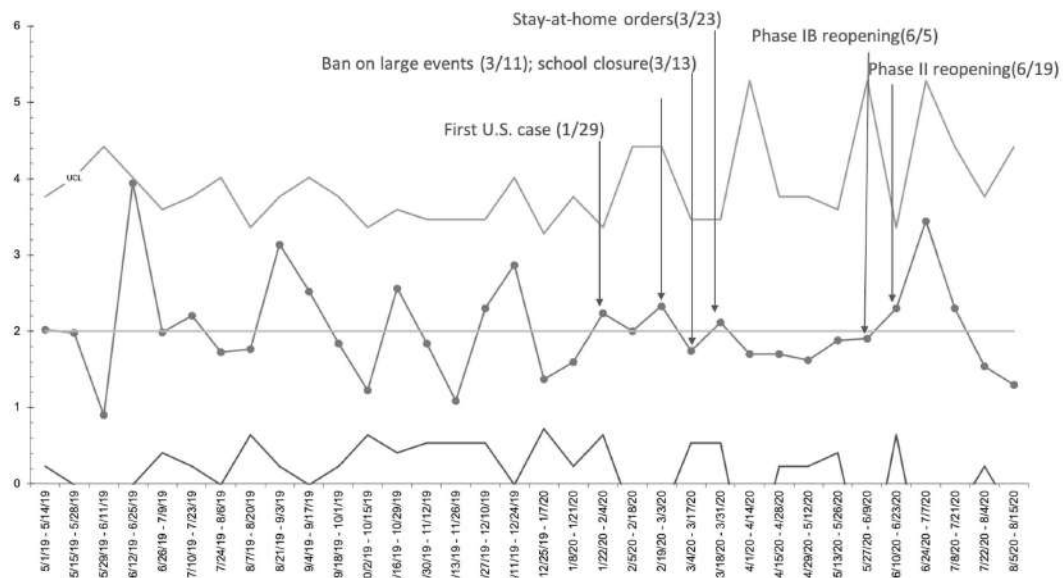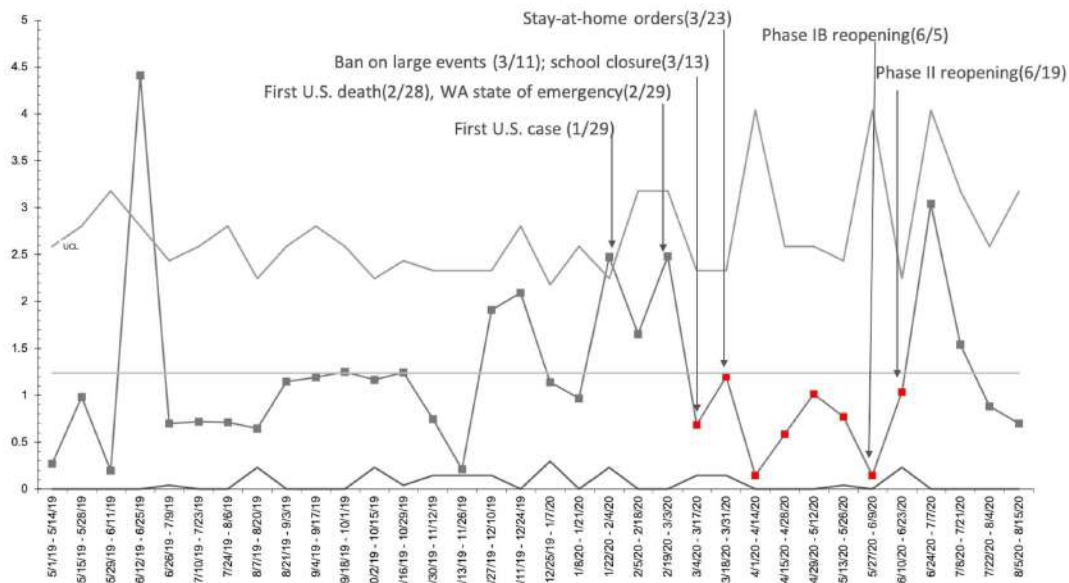

Supplement: Supplementary file 3 [file pqs-7-e502-s003.pdf]
