# Supplementary material for: Impact of the COVID-19 Pandemic on the Severity of Diabetic Ketoacidosis Presentations in a Tertiary Pediatric Emergency Department
Source: Pediatr Qual Saf. 2022 Mar 30;7(2):e502. doi: 10.1097/pq9.0000000000000502 (PMC8970094; doi:10.1097/pq9.0000000000000502)
Supplement: Supplementary file 2 [file pqs-7-e502-s002.pdf]

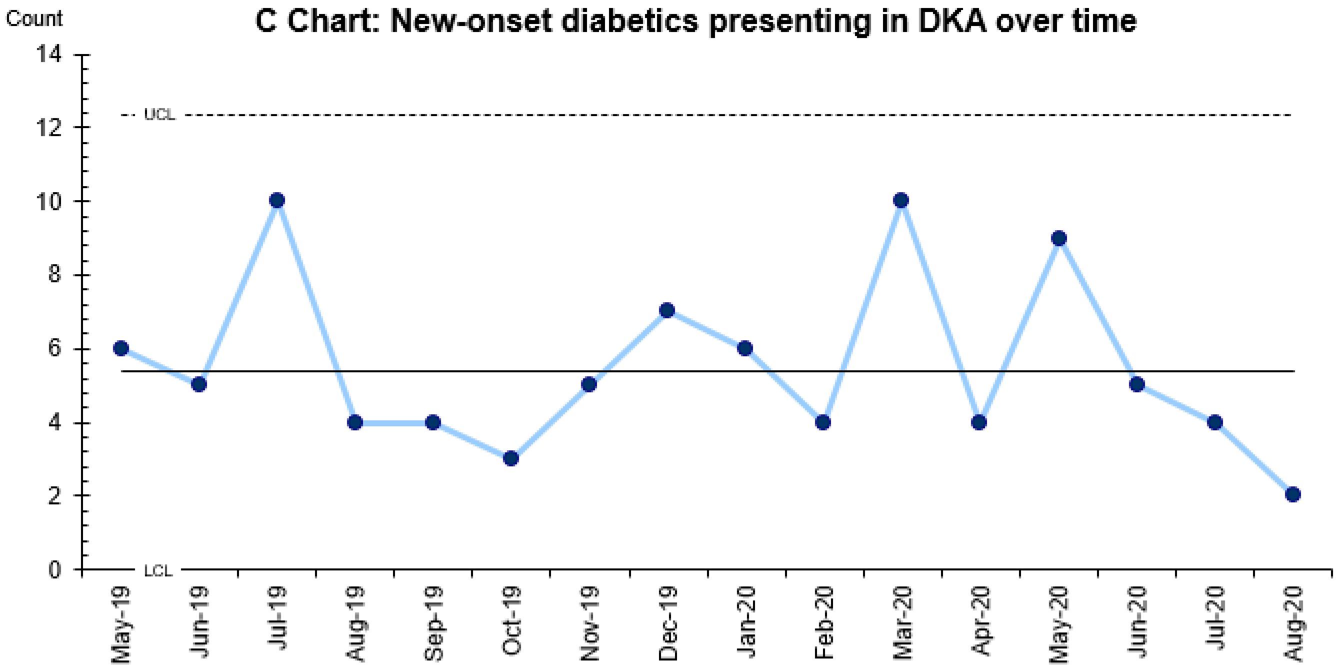
C Chart: New-onset diabetics presenting in DKA over time
Count
14
12
10
8
6
4
2
0
UCL
LCL
May-19
Jun-19
Jul-19
Aug-19
Sep-19
Oct-19
Nov-19
Dec-19
Jan-20
Feb-20
Mar-20
Apr-20
May-20
Jun-20
Jul-20
Aug-20

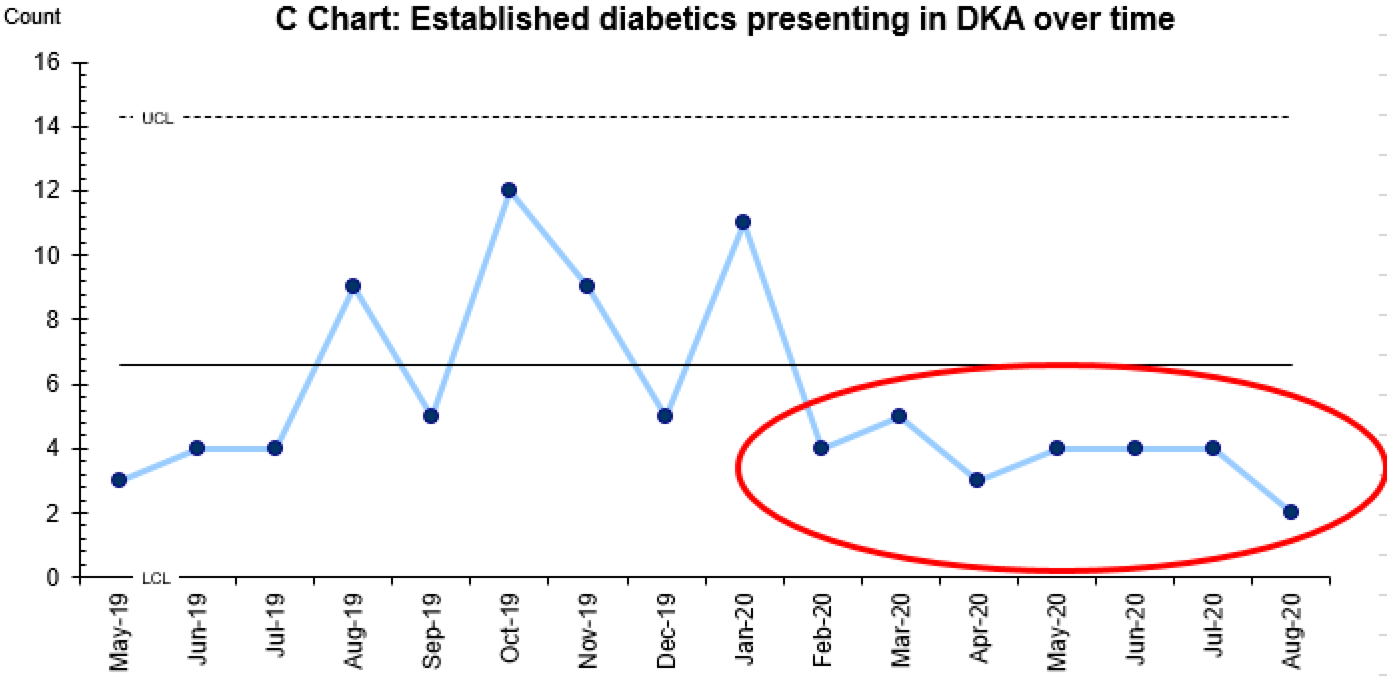
C Chart: Established diabetics presenting in DKA over time
Count
16
14
12
10
8
6
4
2
0
UCL
LCL
May-19
Jun-19
Jul-19
Aug-19
Sep-19
Oct-19
Nov-19
Dec-19
Jan-20
Feb-20
Mar-20
Apr-20
May-20
Jun-20
Jul-20
Aug-20
