# Supplementary material for: Impact of the COVID-19 Pandemic on the Severity of Diabetic Ketoacidosis Presentations in a Tertiary Pediatric Emergency Department
Source: Pediatr Qual Saf. 2022 Mar 30;7(2):e502. doi: 10.1097/pq9.0000000000000502 (PMC8970094; doi:10.1097/pq9.0000000000000502)
Supplement: Supplementary file 4 [file pqs-7-e502-s004.pdf]

Table 3. Secondary outcomes for pediatric patients with new-onset diabetes presenting with DKA

	Pre-COVID-19 (n=53)	COVID-19 (n=34)	All patients (n=87)	P value
PICU admission, n (%)	23 (43.4%)	21 (61.7%)	44 (50.6%)	0.095
Osmotic fluid administration[1], n (%)	2 (3.8%)	3 (8.9%)	5 (5.8%)	0.375
Mechanical ventilation, n (%)	0 (0%)	2 (5.9%)	2 (2.3%)	0.150

[1]Osmotic fluid administration: Mannitol or hypertonic saline

Abbreviations

PICU: pediatric intensive care unit
